# Enhancing peripheral nerve regeneration through NaOH‐based decellularization of human nerve tissue

**DOI:** 10.1002/btm2.70072

**Published:** 2025-09-12

**Authors:** Subin Kim, Seong Hyuk Park, Jiyeon Mun, Soon Won Jung, Won Jai Lee, Dong Won Lee, Kee‐Won Lee

**Affiliations:** ^1^ R&D Center L&C BIO Co., Ltd Seoul Republic of Korea; ^2^ Department of Plastic and Reconstructive Surgery Institute for Human Tissue Restoration, Yonsei University College of Medicine Seoul Republic of Korea

**Keywords:** allograft, decellularization, human nerve, peripheral nerve regeneration, sodium hydroxide

## Abstract

Peripheral nerves are vulnerable to trauma, pressure, and surgical injuries, complicating the regeneration process. While the autograft remains the gold standard for recovery, limitations such as tissue availability and donor site morbidities have led to the exploration of the allografts. However, conventional detergent‐based decellularization methods in preparing allografts often cause residual toxicity and damage to the extracellular matrix (ECM). To address such challenges, we propose a sodium hydroxide (NaOH)‐based decellularization technique that minimizes harmful residues. Our findings demonstrate that this method effectively removes inflammatory materials while preserving the ECM components and structures, and significantly reduces lipid and detergent residues. In vitro studies confirmed that the human nerves processed with the NaOH‐based decellularization technique show low cytotoxicity and support elevated cell viability and proliferation. We further compared the performance of NaOH‐based decellularized human nerves with that of autografts through an in vivo rabbit sciatic nerve defect model. NaOH‐based decellularized nerves showed functional recovery comparable to autografts. Our findings demonstrate structural regeneration through neurofilament and laminin expression, indicating recovery levels similar to those of autografts. This study highlights that decellularized human nerve grafts through the NaOH‐based protocol can promote nerve regeneration comparable to autografts, which can offer a safe and effective option for the treatment and reconstruction of peripheral nerve defects.

**Translational Impact Statement**

This study proposes a novel sodium hydroxide (NaOH)‐based technique for decellularizing human nerve, effectively removing nuclear material while preserving extracellular matrix integrity. This method reduces residual toxicity and supports cell viability and proliferation. Notably, NaOH‐based decellularized human nerve achieves functional and structural recovery comparable to autografts, providing a safe and effective option for reconstructing peripheral nerve defects. These results represent a significant advancement in treatment strategies for nerve injuries, potentially enhancing patient outcomes in both traumatic and reconstructive surgical settings.

## 1 INTRODUCTION

Peripheral nerves extend throughout the body, transmitting sensory and motor signals to the central and peripheral nervous systems. Their widespread distribution makes them vulnerable to injuries, including trauma, iatrogenic injury from tumor resection and radiotherapy, and pressure‐related damage.^1^, ^2^ Managing these injuries is challenging, as outcomes depend on the nerve's location and function. Delayed regeneration can lead to muscular atrophy and neuromuscular junction loss.^2^

A key approach to promoting nerve regeneration is the use of appropriate scaffolds to guide the process. Currently, autografts remain the gold standard for peripheral nerve regeneration.^3^, ^4^ The main advantage of autografts is a well‐preserved internal structure, where the basal lamina, laminin, and neurotrophic factors in the extracellular matrix (ECM) provide an ideal conduit for regeneration.^5^ Additionally, autografts are safe and immune response‐free since they use the patient's own nerves.^5^, ^6^ However, they have several inherent limitations. They are not an option when suitable tissue is unavailable, and obtaining graft material can be difficult, especially for multiple grafting sites. Moreover, donor‐site morbidities, like incisional scars and paresthesia, are unavoidable.^7^, ^8^

To address these issues, alternative materials such as nerve conduits and allografts have been explored. Nerve allografts have been investigated as potential substitutes for autografts.^9^ Previous studies have reported comparable outcomes between nerve allografts and conventional autografts.^10^ More recent studies have shown that decellularizing allografts can reduce immunogenicity while preserving the ECM microstructures that are essential for nerve regeneration.^11^, ^12^, ^13^ However, the effectiveness and safety of decellularized nerve allografts may vary depending on the decellularization protocol used during graft production. Inadequate decellularization can trigger an immune rejection response, while excessive decellularization may damage ECM structures and reduce biocompatibility. Various decellularization techniques─including physical, chemical, and enzymatic approaches such as freeze–thaw cycles, supercritical fluid treatment, enzyme digestion, acids, alkalis, and detergent‐based methods─can be applied to human‐derived tissues to produce suitable decellularized nerve allografts.^14^

Currently, detergent‐based decellularization methods, as proposed by Sondell^15^ and Hudson,^16^ are widely used.^8^, ^9^ For example, Avance® (AxoGen Inc., Alachua, FL, USA), the only FDA‐approved product available in the United States, is commercialized based on the Hudson protocol. This method involves treating tissues with various detergents, such as 0.14% Triton X‐100, 125 mM surfobetaine‐10, and 0.6 mM surfobetaine‐16, multiple times over 7–24 h during the decellularization.^10^ Additionally, the Sondell protocol applies 4% sodium deoxycholate (SDC) for 24 h and 3% Triton X‐100 for 12 h, repeated twice. However, prolonged and excessive use of detergents poses the risk of residual chemical retention, which may damage surrounding cells, tissues, and especially neurons.^17^, ^18^ To mitigate these risks, it is crucial to carefully optimize the decellularization technique by considering factors such as detergent type, exposure duration, and thorough washing procedures to ensure complete removal of residual chemicals while preserving the desired ECM characteristics.^19^

Considering these concerns, the use of alkaline substances has been proposed as an alternative method to enhance safety in the cell removal process. Several studies have demonstrated that solutions with extreme pH values are effective in the decellularization by removing lipids, glycosaminoglycans (GAGs) and DNA.^20^, ^21^, ^22^ For instance, the combination of sodium hydroxide (NaOH) at concentrations up to 1 N, along with sodium chloride (NaCl) and ethylenediaminetetraacetic acid (EDTA), has been shown to effectively decellularize tissues while causing fewer biochemical changes compared to traditional detergent‐based protocols.^23^, ^24^, ^25^, ^26^ However, it should be noted that the proper application and optimization of NaOH for neural tissue decellularization has not yet been fully established. Excessively high pH levels may cause damage to ECM components such as proteins and disrupt the ECM structure, both of which are crucial for cellular regeneration.^21^, ^27^ Optimizing the NaOH‐based process to achieve both complete decellularization and structural stability is considered a critical task in creating favorable environments for nerve regeneration.

To overcome the limitations of previous methods and address these challenges, we propose a synergistic decellularization technique combining SDC and NaOH treatments. By reducing the amount of each reagent, we aim to minimize potential negative effects on ECM components and structures. We hypothesize that, compared with individual treatments, the optimal combination of these two methods will enhance both the effectiveness of nerve regeneration and the safety of processed allograft nerves. To test this hypothesis, we optimized the decellularization process by varying reagent concentrations. We assessed both the effectiveness and safety of the decellularization process through histological analysis, biochemical assays, detergent residue measurements, and in vitro cytotoxicity and cell growth evaluations. Subsequently, we compared the in vivo effectiveness of decellularized nerve grafts with autografts using functional, histological, and immunohistochemical analyses in a rabbit sciatic nerve model.

## 2 MATERIALS AND METHODS

### 2.1 Preparation of the decellularized nerve

Human nerves were obtained post‐surgery at Yonsei Severance Hospital (Seoul, Korea; IRB 4‐20221464). After removing connective tissues, nerves were cut into 2–3 cm segments. NaOH‐based decellularized nerves (N‐DCN) were processed by immersing tissues in isopropyl alcohol (Daejeong Chemicals, Korea) (2 h), rinsing with deionized water (6 h), and treating with 0.25 N NaOH (Sigma‐Aldrich, USA) (8 h). After rinsing, the tissues were treated with 4% SDC (Sigma‐Aldrich) (8 h) and rinsed again. Samples were sterilized using gamma irradiation (25 kGy). To optimize the process, SDC concentrations (4%, 6%, 8%) and NaOH concentrations (0.01, 0.25, 1 N) were tested at different durations (2 and 16 h). The Sondell^15^ and Hudson^16^ protocols served as controls for comparing the safety of the decellularization process.

### 2.2 Characterization of the decellularized nerve

#### 2.2.1 Scanning electron microscopy

Native and decellularized nerves were fixed in Karnovsky's fixative (2% glutaraldehyde, 2% paraformaldehyde in 0.1 M phosphate buffer, pH 7.4) (24 h), washed with phosphate‐buffered saline (PBS), and post‐fixed with 1% osmium tetroxide (2 h). They were dehydrated in an ethanol series (50%–100%) and dried using a critical point dryer (EM CPD300, Leica, Germany). Samples were coated with platinum and examined using a field‐emission scanning electron microscope (Merlin, Zeiss, Germany).

#### 2.2.2 Histology and DAPI staining

Native and decellularized nerves were fixed in 10% buffered formalin (2 h), dehydrated in sucrose solution, embedded in OCT compound (Sakura Finetek, Torrance, CA), and sectioned to 8 μm using a cryostat (CM 1950, Leica). Tissue slides were stained with hematoxylin and eosin (H&E), Masson's Trichrome, and Oil Red O to examine the presence of cells, collagens, and lipids, respectively. Nuclei were stained with 4′,6′‐diamidino‐2‐phenylindole (DAPI, Life Technologies, Carlsbad, CA, USA). DAPI intensity was quantified using ImageJ by measuring the average intensity of 6 regions of interest (ROIs) per sample. Oil Red O quantification was performed by analyzing five ROIs using ImageJ and corrected by background intensity. Slides were observed using a fluorescence microscope (Axio Imager M2, Zeiss, Germany).

#### 2.2.3 Biochemical assay

DNA was extracted^28^ and quantified by digesting lyophilized nerve fragments with tissue lysis buffer (iNtron Biotech, Korea) and proteinase K (ThermoFisher, USA). DNA content was measured using a NanoDrop spectrophotometer (NanoDrop One, ThermoFisher). Total collagen and sulfated GAG (sGAG) content were measured from papain‐digested samples using hydroxyproline assay^29^ and dimethyl methylene blue assay.^30^

### 2.3 Residual detergent measurement

Residual SDC in nerve samples, following the Sondell and our protocols, was quantified by high‐performance liquid chromatography (HPLC, 1260 Infinity II, Agilent, USA) using a C18 column (150 mm × 4.6 mm, 5 μm). The mobile phase was acetonitrile and 0.1% formic acid in water (70:30, v/v), with a flow rate of 1.0 mL/min and column temperature at 30°C. Samples (50 μL) were injected and detected using an evaporative light scattering detector (ELSD) at 35°C. The analysis was performed for 20 min, and results were expressed as the SDC amount (ppm) in each sample.

### 2.4 In vitro cytotoxicity, cell viability, and proliferation of decellularized nerve

#### 2.4.1 In vitro cytotoxicity

In vitro cytotoxicity was assessed following ISO 10993‐5.^31^ Mouse fibroblast cells (L929, Korean Cell Line Bank, Korea) were seeded in 6‐well plates (2 × 10^5^ cells/well) in minimum essential medium (MEM, Lonza, Switzerland) with 10% fetal bovine serum (FBS, ThermoFisher) and 1% penicillin–streptomycin (P/S, ThermoFisher). To investigate the cytotoxicity of the materials, the extraction procedure was performed in the aforementioned medium (37°C, 72 h). Polyethylene film and zinc diethyldithiocarbamate (ZDEC) polyethylene film (Hatano Research Institute, Japan) were used for the negative and positive controls, respectively. For the experimental group, the extraction was carried out using decellularized nerves. The reagent control group was incubated with MEM containing 10% FBS and 1% P/S. After 48 h, cell morphology and viability were assessed under an optical microscope, and ROI analysis was performed to compare cytotoxicity. The ROI was divided into six equal sections using ImageJ, and the average cell count per ROI was calculated.

#### 2.4.2 Cell viability

Human neuroblastoma cells (SH‐SY5Y, Korean Cell Line Bank) were cultured in MEM with 10% FBS and 1% P/S at 37°C, with medium changes every 3 days. Cells were trypsinized at 80% confluence. Cell viability was measured using the Live/Dead Viability/Cytotoxicity Kit (Invitrogen, Carlsbad, CA, USA). Cells were seeded on coverslips at 6 × 10^4^ cells/well, exposed to 70% methanol as a negative control, and incubated with live/dead reagents. After incubation, cells were observed under a fluorescence microscope (Axio Imager M2, Zeiss, Germany). Viability was calculated as the ratio of live‐to‐total cells.

#### 2.4.3 Cell proliferation

Cell proliferation was assessed using the CCK‐8 Assay. Solubilized ECM was obtained by digesting lyophilized nerves with 1 mg/mL pepsin in 0.1 M HCl (72 h, RT) and filtered through a 0.22 μm PES membrane filter (Merck, USA).^32^ Plates were pre‐coated with poly L‐lysine and solubilized ECM from native or decellularized nerves for 24 h. SH‐SY5Y cells (1 × 10^4^ cells/100 μL) were cultured for 1, 2, and 4 days, and CCK‐8 reagent was added. Absorbance at 450 nm was measured using a microplate reader (Varioskan LUX, ThermoFisher).

### 2.5 Animals and surgical procedures

The experimental protocols were approved by the Institutional Animal Care and Use Committee of Yonsei Medical Center (approval number: 2021‐0045), and all procedures followed the ARRIVE (Animal Research: Reporting In Vivo Experiments) guidelines and the institutional ethical standards for animal research. Eighteen male New Zealand White rabbits (15 weeks old, 2.5 kg; DooYeol Biotech, Korea) were randomly assigned to three experimental groups: (a) excised rabbit nerve (Autograft), (b) NaOH‐based decellularized human nerve (N‐DCN), and (c) defect without treatment (Defect) (6 rabbits/group). Male rabbits were used in the study expecting less experimental interference. Based on power analysis for 80% statistical power at alpha 0.05, six animals per group were used to detect statistical significance among different experimental conditions. Three untreated rabbits served as a positive control (Native). Animals were anesthetized with Rompun (5 mg/kg; Bayer, Korea) and ketamine‐HCl (50 mg/kg; Huons, Korea). The 15‐mm segment of the right sciatic nerve was excised, and each group received a different nerve graft before nerve suturing with 4‐0 nylon. In the Autograft, the excised nerves were reversed and reimplanted.

### 2.6 Functional recovery assessment

#### 2.6.1 Motor functional recovery rate

Ankle recovery rate for each experimental group was recorded by lifting the animals and capturing their movements using a digital camera (SELP1650, Sony, Japan). After lifting, the angle between the foot and tibia was measured with the ankle as the reference point. This measurement was converted to a relative value, using 79° as the baseline representing full motor ability. The animals were observed at 1, 6, 12, 18, and 24 weeks post‐implantation.

#### 2.6.2 Ankle stance angle (ASA) measurement

Ankle stance angle (ASA) measurement. Long‐term motor function recovery was assessed using ASA measurements, with the ankle angle changes during walking serving as an indicator of motor performance. To evaluate the walking ability at 24 weeks post‐implantation, animals were guided along a designated walkway, and their movements were recorded using a digital camera (SELP1650). Ankle angles were measured at ground contact and just before lift‐off, and the difference between these two angles was quantified. For each animal, six gait cycles were analyzed (*n* = 6). The average ASA for each group was then calculated and compared.

### 2.7 Muscle volume, muscle weight, and myofilament measurement

To assess muscle mass changes, tissues were explanted 24 weeks post‐implantation and photographed. For weight measurement, the muscles were dried on absorbent paper and weighed using a digital balance (2241‐1S, Sartorius AG, Germany). For volume measurement, the muscles were immersed in a test tube containing 30 mL of PBS, and the increased PBS volume was measured. Myofiber morphology was examined by cross‐sectioning, creating 4‐μm paraffin slides, and performing H&E staining.

### 2.8 Myelin regeneration measurement

To assess myelin regeneration, explanted tissues were fixed in 2% glutaraldehyde‐2% paraformaldehyde (Sigma‐Aldrich). Tissues were washed and postfixed with 1% osmium tetroxide (Sigma‐Aldrich), dehydrated with graded ethanol (50%–100%), and infiltrated with propylene oxide. Specimens were embedded in Poly/Bed 812 (Polysciences), polymerized in an electron microscope oven (TD‐700, DOSAKA, Japan) (65°C, 12 h), and sectioned into 200‐nm semi‐thin slices using an ultra‐microtome (EM UC7, Leica). Myelin was observed with toluidine blue staining under an optical microscope. Sections were re‐cut to 80 nm and double‐stained with uranyl acetate and lead citrate, then imaged using TEM (JEM‐1011, JEOL, Japan). Myelinated nerves were counted from six nerves per group, and myelin sheath thickness and nerve diameter were measured from a single nerve in each group (*n* = 20).

### 2.9 Immunohistochemistry

At 24 weeks post‐implantation, explanted tissues were fixed in 10% formalin, embedded in paraffin, and sectioned at 4 μm with a microtome (RM2235, Leica). Axon regeneration was assessed using immunohistochemistry for NF200 and laminin with anti‐NF200 (N0142, Sigma‐Aldrich), anti‐laminin (NB300‐144, Novus, USA), and the REAL EnVision™ Detection System (Dako, Denmark). Image analysis was performed with ImageJ, applying ROI and spatial calibration. ROIs for counting were six equal sections, with an RGB histogram threshold used to measure the area‐to‐area ratio of the brown stain.

### 2.10 Statistical analysis

Data were expressed as the mean ± standard deviation. Statistical analysis was performed using Prism version 6 (GraphPad Software, La Jolla, CA, USA). All experiments included at least three independent replicates. Statistical significance was analyzed using the Student's *t*‐test and one‐way analysis of variance (ANOVA) with a Bonferroni post‐hoc test for the comparison of two groups and of more than two groups, respectively. A *p*‐value of less than 0.05 was considered statistically significant.

## 3 RESULTS

### 3.1 Optimization and characterization of the NaOH‐based decellularized nerve

**FIGURE 1:**
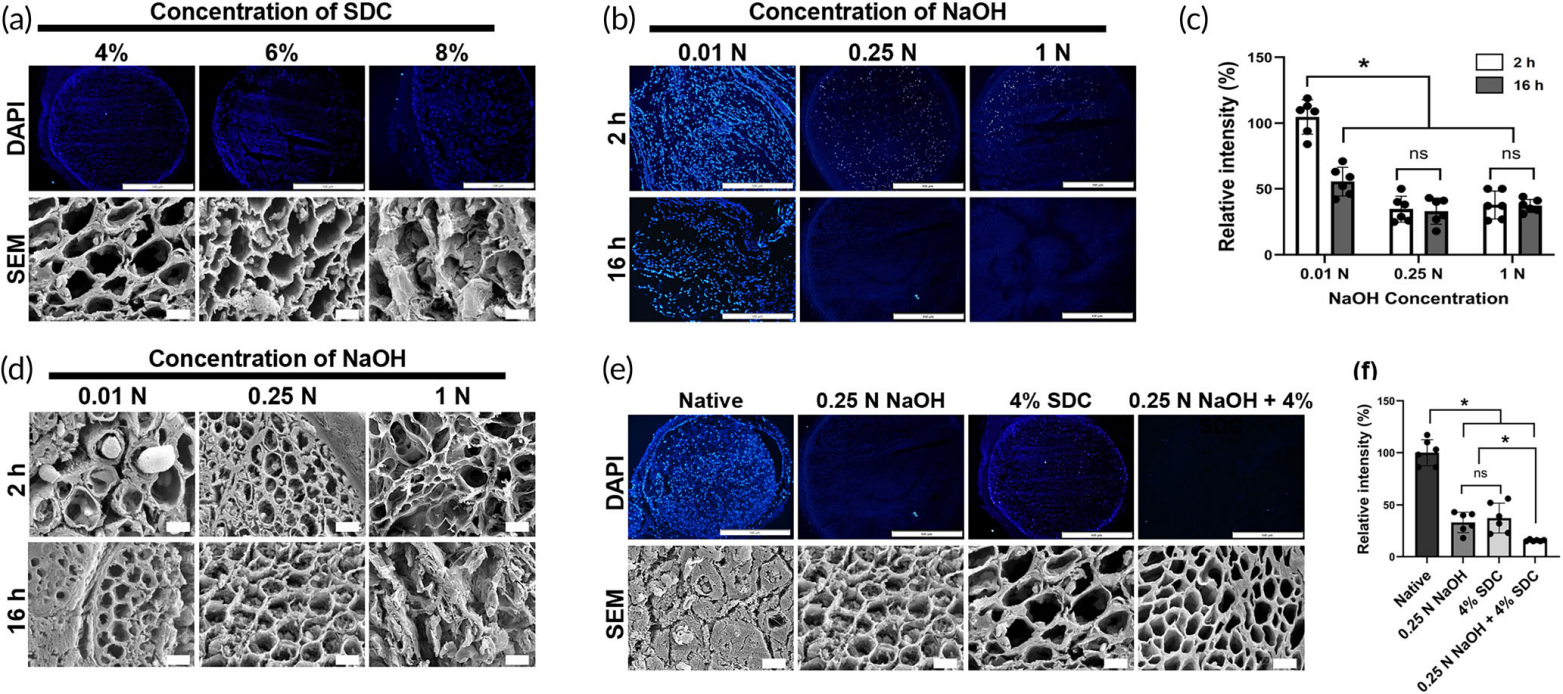
Optimization of decellularization. (a) Representative DAPI staining and SEM images of decellularized nerves treated with various concentrations of SDC for 2 h. Magnification = 200× (top row) and 2000× (bottom row). Scales bars = 100 μm (top row) and 2 μm (bottom row). (b) Representative DAPI staining images of decellularized nerves treated with various concentrations of NaOH for 2 and 16 h. Magnification = 200×. Scale bars = 100 μm. (c) Quantification of the relative intensity of DAPI staining in each group. Data are presented as mean ± SD (*n* = 6). * indicates *p* < 0.05. (d) Representative SEM images of decellularized nerves treated with various concentrations of NaOH for 2 and 16 h. Magnification = 100×. Scale bars = 10 μm. (e) Representative DAPI staining and SEM images of decellularized nerves treated with NaOH for 16 h and SDC for 2 h. Magnification = 200× (top row) and 2000× (bottom row). Scales bars = 100 μm (top row) and 2 μm (bottom row). The images presented in (e) (4% SDC, 0.25 N NaOH) are respectively identical to those obtained under the optimized experimental conditions shown in (a) (4% SDC) and (d) (0.25 N NaOH, 16 h). (f) Quantification of the relative intensity of DAPI staining in each group. Data are presented as mean ± SD (*n* = 6). * indicates *p* < 0.05.

We first examined the concentration and treatment time‐dependent effects of SDC and NaOH on the decellularization and ECM structures of allograft nerves. DAPI staining and scanning electron microscopy (SEM) revealed that SDC treatment above 6% for 6 h damaged the nervous ECM structure (Figure 1a). In the case of NaOH‐only treatments, complete cell removal was not achieved even at 1 N when treated for 2 h. However, extending the treatment to 16 h at concentrations of 0.25 N resulted in clear decellularization (Figure 1b,c), while preserving the ECM structure (Figure 1d). Under the aforementioned treatment conditions of 16 h at concentrations of 0.25 N, the pH level increased from neutral to approximately pH 10. The pH subsequently returned to a neutral level after the final washing process (Figure S1). Taken together, the optimized condition for both cell removal and ECM preservation was established as 0.25 N NaOH and 4% SDC treatment for 16 h (Figure 1e,f).

### 3.2 Characterization of the decellularized nerve

**FIGURE 2:**
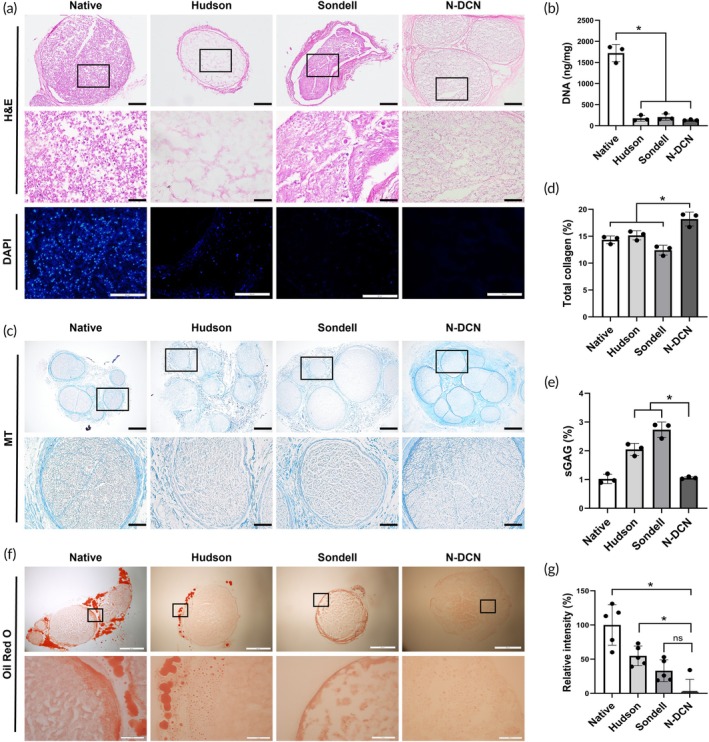
Characterization of decellularized nerves. (a) Representative H&E and DAPI staining images of native and decellularized nerves. Magnification = 40× (upper row), 200× (middle row), and 400× (lower row). Scales bars = 500 μm (upper row), 100 μm (middle row), and 50 μm (lower row). (b) Quantification of DNA in native and decellularized nerves using biochemical assays. Data are presented as mean ± SD (*n* = 3). * indicates *p* < 0.05. (c) Representative Masson's Trichrome staining images of native and decellularized nerves. Magnification = 40× (upper row), 200× (lower row). Scales bars = 500 μm (upper row), 100 μm (lower row). (d, e) Quantification of total collagen and sGAG content in native and decellularized nerves using biochemical assays. Data in (d, e) are presented as mean ± SD (*n* = 3). * indicates *p* < 0.05. (f) Representative Oil Red O staining images of native and decellularized nerves. Magnification = 40× (top row) and 200× (bottom row). Scales bars = 500 μm (top row) and 100 μm (bottom row). (g) Quantification of the relative intensity of Oil Red O staining in each group, analyzed using Image J software. Data are presented as mean ± SD (*n* = 5). * indicates *p* < 0.05.

To characterize the decellularized nerve established the optimization above, histological and biochemical analyses were performed. H&E and DAPI staining showed the removal of nuclear components by comparing the NaOH‐based decellularized nerve (N‐DCN) with native nerve (Native), the Hudson protocol group (Hudson), and the Sondell protocol group (Sondell) (Figure 2a). Quantification of residual DNA showed that N‐DCN contained significantly lower residual DNA content (134.44 ± 13.93 ng/mg) compared to the Native (1722.43 ± 204.76 ng/mg; *p* < 0.05) and was similar to Sondell (176.56 ± 68.46 ng/mg) and Hudson (205.01 ± 75.47 ng/mg) (Figure 2b).

Masson's Trichrome staining revealed the presence of collagen deposition in each group. We confirmed that N‐DCN shows greater collagen deposition compared to Hudson and Sondell (Figure 2c). Total collagen content was significantly higher in the N‐DCN (18.20% ± 1.29%) compared to Native (14.34% ± 0.72%; *p* < 0.05), Sondell (12.40% ± 0.91%), and Hudson (15.16% ± 0.84%) (Figure 2d). The sGAG content was similar in the N‐DCN (1.06% ± 0.04%) and Native (1.02% ± 0.16%; *p* > 0.05) (Figure 2e), but lower than Sondell (2.74% ± 0.27%) and Hudson (2.05% ± 0.21%).

Oil Red O staining showed lower lipid content in the N‐DCN compared to other groups. The stained area of lipid in the N‐DCN (1% ± 17%; *p* < 0.05) was significantly smaller than in the Native (100% ± 27%; *p* < 0.05) and the Hudson (55% ± 12%; *p* < 0.05), and statistically comparable to that of Sondell (33% ± 14%; *p* = 0.1603) (Figure 2f,g).

### 3.3 Residual detergent of the NaOH‐based decellularized nerve

**FIGURE 3:**
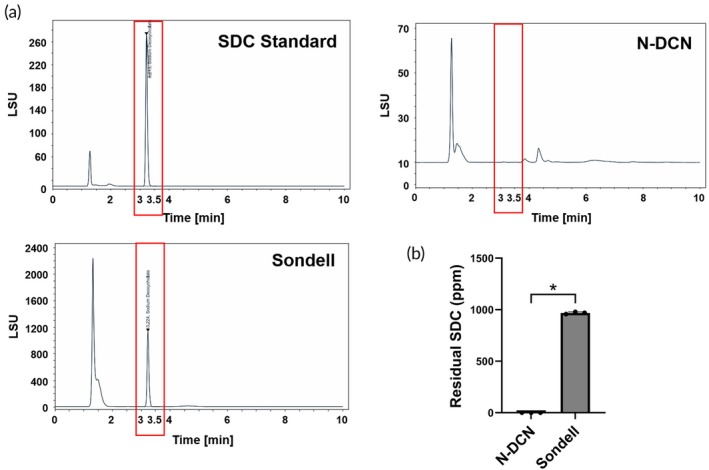
Residual detergent of the decellularized nerve. (a) Residual SDC in Sondell protocol and N‐DCN measured using HPLC. (b) Quantification of SDC remaining in each sample unit. Data are presented as mean ± SD (*n* = 3). * indicates *p* < 0.05.

If detergent residues remain in the decellularized nerve, they may cause damage to the implantation area and surrounding tissues. Therefore, we quantified the residual detergent levels in the decellularized nerve using HPLC. Based on the calibration results, SDC showed a distinct peak with a retention time between 3 and 3.5 min. Residual SDC was detected only in the Sondell, while none was detected in the N‐DCN (Figure 3).

### 3.4 In vitro cytotoxicity, cell viability, and proliferation of the decellularized nerve

**FIGURE 4:**
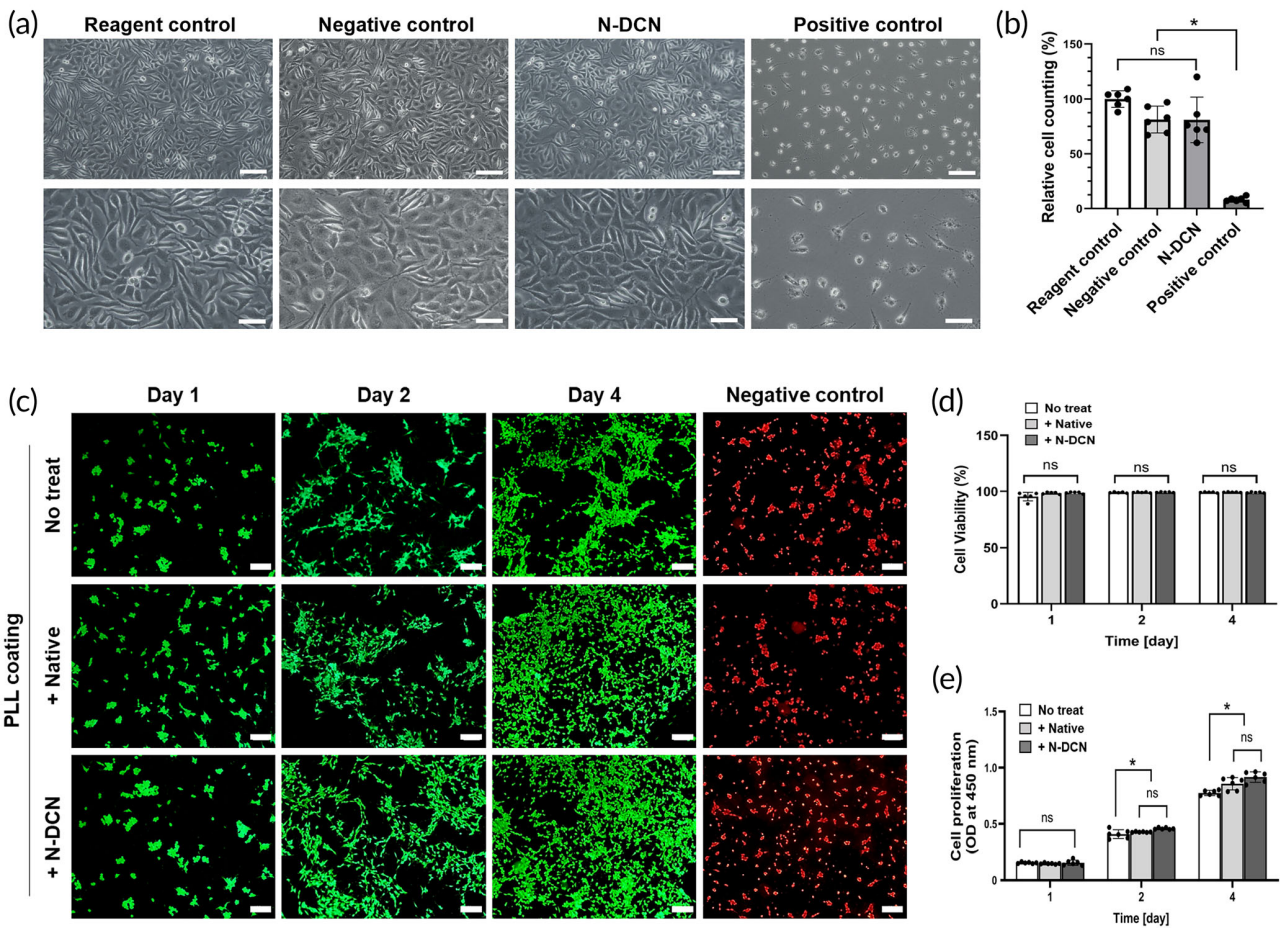
In vitro cytotoxicity, cell viability and proliferation of the decellularized nerve. (a) Representative images of L929 fibroblasts after 48 h of incubation with extracts of controls and N‐DCN following the ISO 10993‐5 protocol. Magnification = 100× (top row) and 400× (bottom row). Scales bars = 100 μm (top row) and 50 μm (bottom row). (b) Relative cell counting in each group. Data are presented as mean ± SD (*n* = 6). * indicates *p* < 0.05. (c) Representative Live/Dead staining images of SH‐SY5Y cells after 1, 2, and 4 days of incubation following coating with ECM from each group. Green indicates live cells and red indicates dead cells. Magnification = 200×. Scale bar = 50 μm. (d) Cell viability in each group. Data are presented as mean ± SD (*n* = 5) and revealed no significant differences between groups. (e) Cell proliferation after 1, 2, and 4 days of incubation following coating with ECM from each group, measured using the CCK‐8 assay. Data are presented as mean ± SD (*n* = 6). * indicates *p* < 0.05.

To comprehensively demonstrate the safety mentioned above, we evaluated the cytotoxicity, cell viability, and proliferation of the decellularized nerves in vitro. The in vitro cytotoxicity test demonstrated that the cell morphology treated with N‐DCN extracts was similar to that treated with the reagent control extract. No rounded cells or loose attachments were observed. The relative cell counting of the N‐DCN treated group was 91%, corresponding to grade 1 (>80%) viable cells (Figure 4a,b). SH‐SY5Y cells seeded on ECM of N‐DCN showed a similar level of cell viability (Figure 4c,d) and proliferation (Figure 4e) to that treated with the reagent control extract.

### 3.5 In vivo functional recovery assessment

**FIGURE 5:**
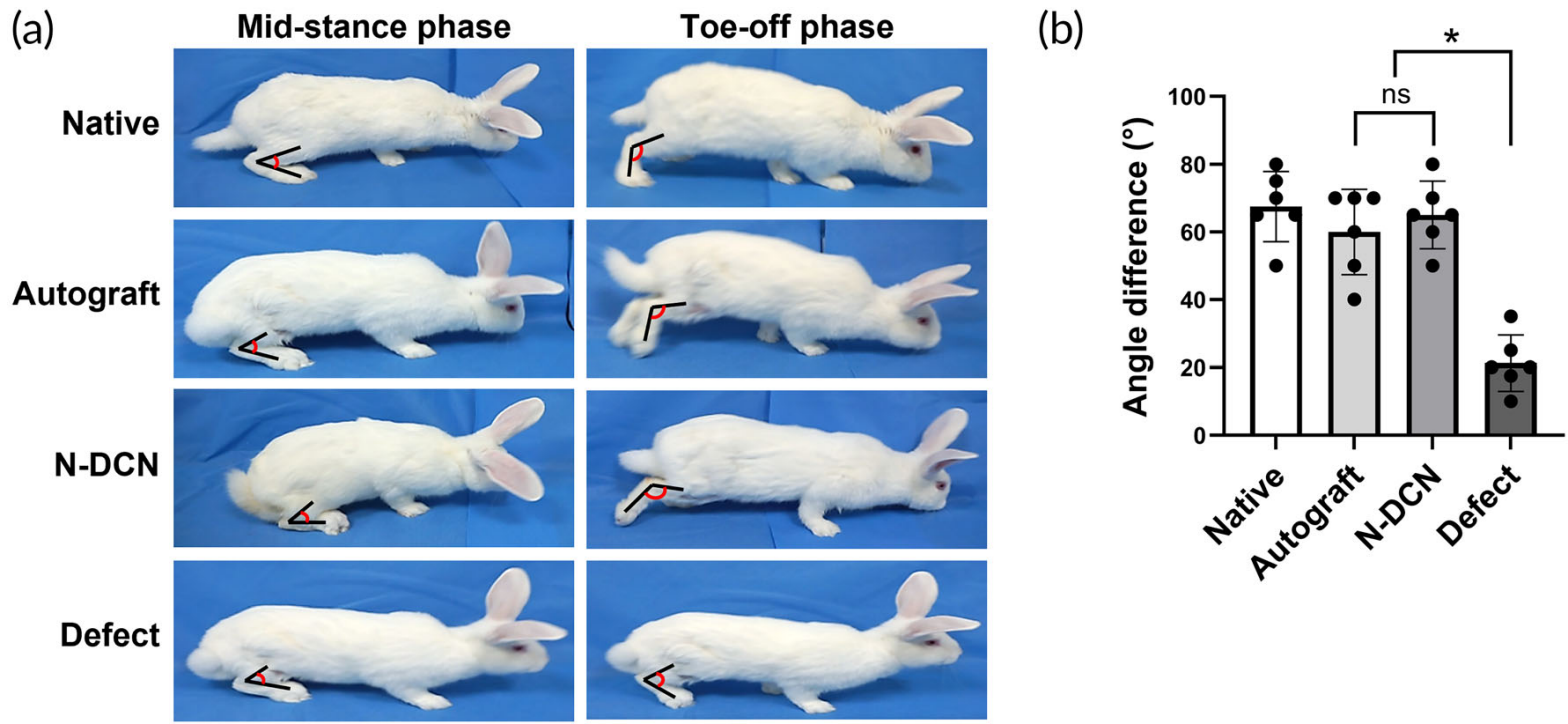
In vivo functional analysis. (a) Representative images of gait analysis at 24 weeks post‐implantation. (b) Quantification of the angle difference between the mid‐stance and the toe‐off phases. Data are presented as mean ± SD (*n* = 6). * indicates *p* < 0.05.

To assess the functional recovery of the decellularized nerve, we measured ASA through gait analysis post‐implantation. For ASA measurement, the hind ankle angle was recorded at the mid‐stance and toe‐off phases, and the differences between the groups were compared (Figure 5a, Movies S1–S4). The angle difference between the mid‐stance and toe‐off phases was 64.5° ± 2.5° for the Native, 26° ± 8.9° for the Defect, and 60° ± 12.2° and 61.5° ± 5° for the Autograft and N‐DCN, respectively; this indicates that ankle movement in both the Autograft and N‐DCN was significantly greater (*p* < 0.05) than in the Defect (Figure 5b).

Motor functional recovery rate was evaluated by measuring the angle between the foot and tibia, using the ankle as the reference point (Figure S2a). At 18 weeks post‐implantation, the N‐DCN showed signs of recovery, with four subjects achieving complete recovery and two showing partial improvement (Figure S2b). The Autograft exhibited a similar recovery pattern, with four subjects achieving complete recovery and two showing partial recoveries by 24 weeks. The number of recovered subjects was significantly higher (*p* < 0.05) in both the N‐DCN and Autograft compared to the Defect.

### 3.6 Immunohistochemical analysis in early stage

To evaluate the early stage of nerve regeneration, we proceed with additional in vivo experiments. Further details on the additional experiments are provided in the Supporting Information. Surgical procedures were described in Figure S3. Tissue responses were assessed through immunohistochemistry. Primary antibodies and detection kits used in immunohistochemistry are listed in Table S1. The N‐DCN showed no significant differences in CD68^+^ cells compared to the other groups (Figure S4). N‐DCN exhicbited a significant increase in CD31^+^ cells relative to the Defect (*p* < 0.05), with levels comparable to those of Autograft and Hudson. N‐DCN showed a significantly increased collagen type I area compared to all other groups (*p* < 0.05, Figure S5).

### 3.7 Muscle volume, muscle weight, and myofilament measurement

**FIGURE 6:**
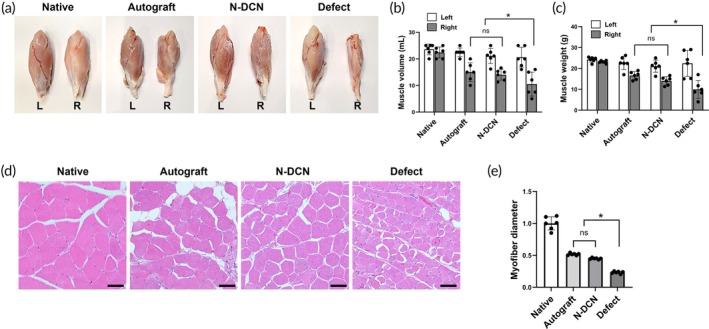
Muscle volume, weight, and myofilament measurements. (a) Representative images of muscles from each group after harvest at 24 weeks post‐implantation. L, left (untreated); R, right (treated). (b) Measurements of harvested muscle volume and (c) muscle weight. Data are presented as mean ± SD (*n* = 6). * indicates *p* < 0.05. (d) Representative H&E staining images of each group at 24 weeks post‐implantation. Magnification = 400×. Scale bar = 50 μm. (e) Relative myofiber diameter of each group. Data are presented as mean ± SD (*n* = 6). * indicates *p* < 0.05.

To confirm motor function recovery from a muscular perspective, we assessed the muscles connected to the implanted nerve through gross morphological and histological analyses. Gross morphological examination indicated that the degree of muscle atrophy in the N‐DCN was comparable to that in the Autograft and superior to that in the Defect (Figure 6a). The Defect showed the largest reduction in muscle mass (weight reduction: 56% ± 0.12%, volume reduction: 49% ± 0.08%). The Autograft lost 27% ± 0.06% of its muscle weight and 33% ± 0.07% of its volume, while the N‐DCN lost 30% ± 0.06% of its muscle weight and 25% ± 0.01% of its volume. Compared with the Defect, both groups showed a significantly smaller decrease (*p* < 0.05) in postoperative muscle mass (Figure 6b,c, Tables 1 and 2). H&E staining of the harvested muscles showed similar results (Figure 6d) with the Defect having significantly fewer myofibers and shorter (*p* < 0.05) myofiber diameters than those in the Autograft and N‐DCN (Figure 6e).

**TABLE 1:** Muscle volume.

	Muscle volume (mL)			
	Native	Autograft	N‐DCN	Defect
Left	23.6 ± 0.5	22.5 ± 1.8	19.2 ± 0.9	20.6 ± 3.7
Right	22.4 ± 0.4	15 ± 1.8	14.3 ± 0.2	10.5 ± 3.2
Loss (%)	8 ± 0.03	33 ± 0.07	25 ± 0.01	49 ± 0.08

**TABLE 2:** Muscle weight.

	Muscle weight (g)			
	Native	Autograft	N‐DCN	Defect
Left	24.2 ± 0.5	22.6 ± 3.2	21.1 ± 2.8	22.4 ± 6.2
Right	23.1 ± 0.4	16.6 ± 1.9	14.1 ± 2.1	9.9 ± 4.3
Loss (%)	5 ± 0.02	27 ± 0.06	30 ± 0.06	56 ± 0.12

### 3.8 Myelin regeneration assessment

**FIGURE 7:**
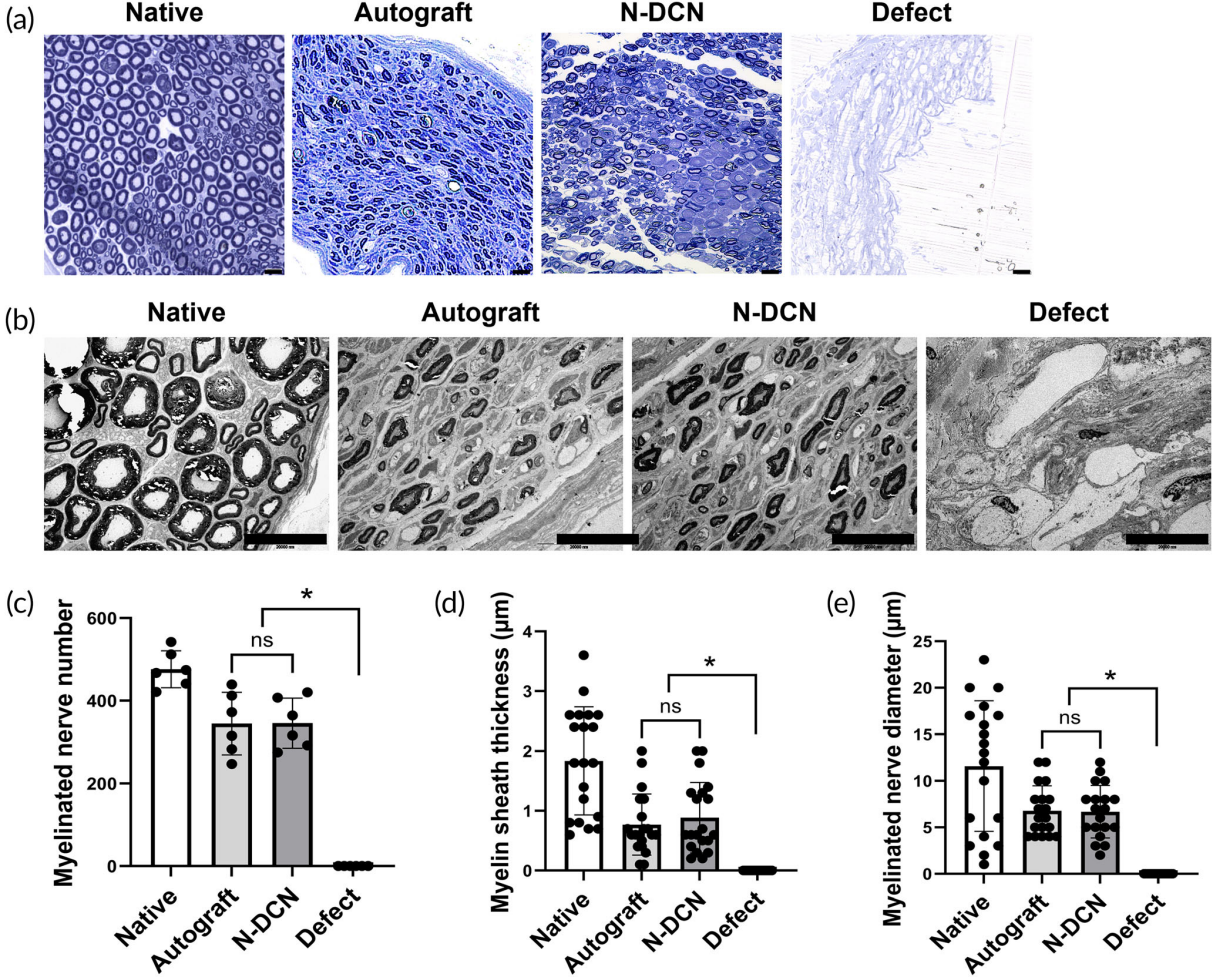
Myelin regeneration assessment. (a) Representative toluidine blue staining images of harvested nerves from each group. Magnification = 400×. Scale bar = 20 μm. (b) Representative TEM images of harvested nerves from each group. Magnification = 2000×. Scale bar = 2 μm. (c) Quantification of the number of myelinated nerves, (d) myelin sheath thickness, and (e) myelinated nerve diameter. Data in (c) are presented as mean ± SD (*n* = 6) and data in (d, e) are presented as mean ± SD (*n* = 20). * indicates *p* < 0.05.

To compare the extent of nerve regeneration from a histological perspective, we evaluated changes in myelination—a key indicator of nerve regeneration—using toluidine blue staining and TEM. The N‐DCN harvested 24 weeks after grafting showed axonal regeneration with myelination. The diameter, thickness, and number of myelinated nerve fibers did not differ between the Autograft and N‐DCN, whereas the Defect showed fibrous tissue with no myelination (Figure 7a,b). Myelination quantification indicates equivalence between the Autograft and the N‐DCN, with a significant difference compared to the Defect (*p* < 0.05) (Figure 7c–e).

### 3.9 Nerve regeneration assessment

**FIGURE 8:**
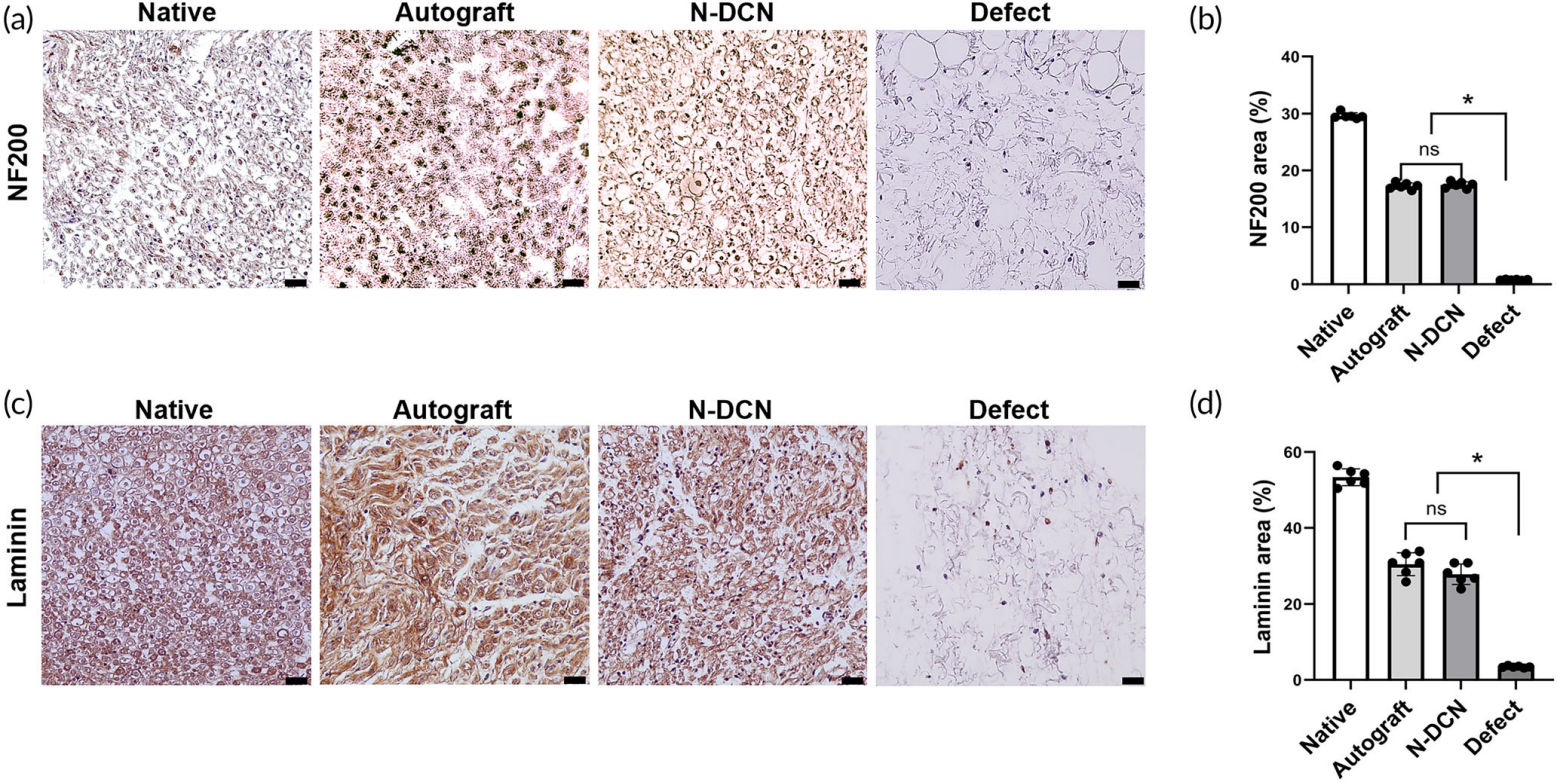
Nerve regeneration assessment. (a) Representative images of immunohistochemistry staining of NF200 in each group at 24 weeks post‐implantation. Magnification = 400×. Scale bar = 50 μm. (b) Quantification of NF200‐stained areas in each group (*n* = 6). * indicates *p* < 0.05. (c) Representative images of immunohistochemistry staining of laminin in each group at 24 weeks post‐implantation. Magnification = 400×. Scale bar = 50 μm. (d) Quantification of laminin‐stained areas in each group (*n* = 6). * indicates *p* < 0.05.

To elucidate nerve regeneration at the molecular level, we evaluated the expression of neurofilament and laminin—major intracellular structural components and markers of nerve regeneration—using immunohistochemistry. Immunohistochemical staining showed that the regeneration of neurofilament and laminin structures in N‐DCN was comparable to that in Autograft (Figure 8a,c). Neurofilament and laminin expression areas of N‐DCN (17.44 ± 0.59, 27.89 ± 2.71) were similar to those in the Autograft (17.25 ± 0.58, 30.53 ± 3.01), while the Defect (3.45 ± 0.24, 0.81 ± 0.08) showed significantly decreased (*p* < 0.05) expression (Figure 8b,d).

## 4 DISCUSSION

In this study, we aimed to demonstrate that a NaOH‐based protocol effectively decellularizes human nerves and enhances peripheral nerve regeneration. An effective decellularization protocol should eliminate immunogenic components, such as DNA, lipids, and membrane proteins, while preserving the ECM microstructure.^33^, ^34^, ^35^ Commonly used methods, such as the Sondell protocol (using Triton X‐100 and SDC in four steps) and the Hudson protocol (utilizing surfobetaine‐10 and surfobetaine‐16, also in four steps), typically employ multiple detergents over several steps and an extended timeframe.

Notably, our findings indicate that NaOH can serve as a complementary agent to detergents in the decellularization process. It is well known that detergents facilitate cell removal by disrupting the cell and nuclear membranes due to their inherent chemical specificity.^14^ However, given the chemical mechanism of detergents in cell removal, the difficulty in completely eliminating the residual detergents from complex biological structures, and their undesired effects on the biological materials, minimizing potential risks including cytotoxicity and inflammatory responses and replacing detergents with safer alternatives must be considered to improve both the safety and the performance of graft materials.^36^ As an alternative, NaOH has been previously used to decellularize xenograft heart valves, resulting in improved decellularization and fewer biochemical modifications compared to conventional detergent‐based protocols.^20^, ^21^, ^22^, ^23^, ^24^, ^25^, ^26^ However, the application of NaOH for decellularizing nerve grafts for axonal regeneration has not yet been explored. Compared to conventional methods, our findings highlight the safety and effectiveness of the NaOH‐based decellularization method for processing nerve grafts. This study demonstrates that, even with reduced detergent quantities, process durations, and complexity, the neural structure remained intact and the immunogenic components such as nuclei and lipids were effectively removed. Supporting evidence includes SEM images as well as DAPI and Oil Red O staining results. Taken together, these findings indicate that the NaOH‐based decellularized nerves are safe and biocompatible.

Furthermore, in vitro results suggest that the ECM of decellularized nerves promotes the growth of neuron‐like cells, with no significant difference compared to native nerve tissue. SH‐SY5Y, a neuroblastoma cell line widely used in neuroscience studies,^37^, ^38^, ^39^ was selected to evaluate early neuron proliferation prior to full differentiation. The healthy state of ECM‐treated SH‐SY5Y cells demonstrates the biological safety of the decellularization process. Future studies will investigate how the ECM of decellularized nerve influences differentiation and neurite outgrowth.

Another notable finding related to the NaOH‐based decellularized nerves is their potential for promoting nerve regeneration. Collagen is a critical component in nerve regeneration,^40^ as it regulates Schwann cells and facilitates nerve fiber growth. Preserving total collagen in decellularized nerve tissue has been shown to support this regenerative process.^41^, ^42^

In this study, we observed a relative increase in the collagen‐to‐total weight ratio following decellularization. Although the relative increase is likely due to the removal of cellular components, such as lipids, proteins, and other cytoplasmic materials, the results shown in Figure 2d reveal that the N‐DCN samples possess a higher potential to contain higher contents of collagen after processing. While sGAG is also associated with nerve regeneration, it is a part of chondroitin sulfate proteoglycan (CSPG), which is known to inhibit axonal regeneration.^41^, ^42^ CSPG consists of proteoglycans bound to a protein core, creating both chemical and physical barriers to axonal growth. In this study, sGAG content was preserved; however, due to lipid removal, we expect that sGAG levels may be maintained or reduced in the decellularized nerve. Therefore, our decellularization protocol appears to support collagen preservation, which is beneficial for nerve regeneration, while potentially maintaining or reducing CSPG, a component that hinders regeneration. These findings suggest that the neuro‐friendly properties of NaOH‐based decellularized nerves, including the preservation of collagen and the potential reduction of inhibitory components like CSPG, could substantially strengthen their ability to promote nerve regeneration.

Conventional detergent‐based decellularization methods carry a risk of cytotoxicity due to residual detergents. However, our in vitro results confirmed the safety of the NaOH‐based decellularized nerve and its potential for clinical application. In this study, no residual SDC was detected in the decellularized nerve, whereas it was present in the Sondell protocol. This absence of residual SDC alleviates safety concerns. Additionally, Oil Red O staining demonstrated significant lipid removal from the decellularized nerve, suggesting a reduced potential for immune response upon implantation.

Based on the aforementioned evidence, the potential of NaOH‐based decellularized nerves to promote nerve regeneration has been validated through in vivo animal experiments. To evaluate the effectiveness of nerve regeneration in NaOH‐based decellularized nerves, we assessed motor functional recovery using the ASA,^43^, ^44^ which measures the ankle joint angle during the mid‐stance phase of the gait cycle to evaluate the functional recovery of the sciatic nerve. This measurement provides a more multifaceted and continuous analysis of sciatic nerve recovery than the Sciatic Function Index (SFI).^45^, ^46^ Notably, our findings indicate that the NaOH‐based decellularized nerves support motor functional recovery, suggesting reinnervation.

In addition, early stage observations revealed that NaOH‐based decellularized nerves demonstrated improved regenerative potential and comparable safety compared to Autograft and commercially established methods such as Hudson. The inflammatory response tended to increase up to 1 week; this trend was consistent with previous findings on decellularized nerve grafts.^47^ The N‐DCN elicited an inflammatory response comparable to the Autograft, but lower than that observed with the Hudson. In particular, analyses of neovascularization (nutrient and cellular influx)^48^ and collagen deposition (structural ECM restoration)^49^ revealed that NaOH‐based decellularized nerves exhibited favorable regeneration trends compared to both Autograft and Hudson.

Additionally, TEM and histological analyses support the notion that NaOH‐based decellularized nerves restore sciatic nerve function through remyelination, which is critical for axon regeneration.^50^, ^51^ To confirm structural nerve regeneration at the molecular level, we quantified the areas of neurofilament and laminin, which are known to be the key structural markers of the axons and the ECM, respectively.^52^, ^53^ Neurofilament proteins are the key structural components of the neurons that help maintain their shape and stability.^54^, ^55^, ^56^ Laminin has been reported to play a crucial role in creating a supportive environment for nerve regeneration by promoting cell adhesion, migration, and axonal growth.^57^, ^58^, ^59^ The increasing trends in these two markers provide compelling evidence that supports the previously observed functional and histological recovery, thereby suggesting the restoration of both nerve cells and surrounding nerve structures.^60^

Our study was conducted using rabbits, as they can offer a more suitable model compared to rodents, while being more accessible and cost‐effective than large animals. Further trials are necessary to assess potential human applications. We also validated effectiveness only for moderate gaps (1.5 cm), meaning that results may differ for larger defects and more severe injuries. In the present work, we confirmed the functional recovery through the behavioral analysis, as the assessment of final motor function recovery is thought to be sufficient from a comprehensive regeneration perspective. Although electrophysiological tests could be a valuable tool for evaluating the function of individual nerves, there are instances to clarify that electrical signals can be detected without corresponding overall actual functional recovery.^61^ Therefore, observing actual functional recovery is deemed more suitable than measuring electrical signals in terms of evaluating comprehensive regeneration.

In future studies, the study aims to evaluate the performance and stability of the optimized products by comparing them with those produced using conventional experimental methods. Methodologically, we plan to combine electrophysiological and behavioral analyses to quantitatively assess nerve regeneration in sensory and motor nerves as well as their innervated muscles. Additionally, through the co‐staining of myelin sheath and Schwann cells, we intend to provide a more detailed depiction of the morphological interactions between peripheral nerves and their surrounding structures. Also, further research is needed to confirm the clinical applications and range of indications for both autografts and decellularized nerve grafts.

## 5 CONCLUSIONS

In summary, we introduced a NaOH‐based decellularization protocol for human nerve tissues and validated its safety and effectiveness in enhancing nerve regeneration. By optimizing the protocol with varying durations and concentrations of SDC and NaOH, we effectively removed lipids and cells while preserving ECM structures. The decellularized nerves retained key ECM components essential for nerve regeneration and contained negligible residual detergents, thereby minimizing potential clinical risks. We also demonstrated excellent cytocompatibility of the NaOH‐based allograft nerve and evaluated its effects on nerve regeneration in a rabbit sciatic nerve defect model over 24 weeks. Notably, the decellularized nerves achieved functional and structural recovery comparable to that of autografts. These findings suggest that allograft nerves processed through the NaOH‐based decellularization protocol are promising substitutes for autografts in treating segmental peripheral nerve defects. Taken together, these results highlight the potential of the NaOH‐based decellularization process to overcome the limitations of detergent‐based protocols and to serve as a safer, more biocompatible, and more effective alternative.

## AUTHOR CONTRIBUTIONS

**Subin Kim**: Methodology; data curation; investigation; formal analysis; visualization; writing—original draft; review and editing. **Seong Hyuk Park**: Investigation; writing—original draft; review and editing. **Jiyeon Mun**: Methodology; data curation; investigation; formal analysis; visualization; writing—original draft; review and editing. **Soon Won Jung**: Data curation; investigation; formal analysis; visualization; writing—original draft. **Won Jai Lee**: Investigation; writing—review and editing. **Dong Won Lee**: Conceptualization; methodology; investigation; supervision; resources; writing—review and editing. **Kee‐Won Lee**: Conceptualization; methodology; investigation; validation; supervision; funding acquisition; project administration; writing—original draft; review and editing.

## CONFLICT OF INTEREST STATEMENT

The authors declare no conflicts of interest.

## Supporting information


**Table S1.** Primary antibodies and detection kits used in immunohistochemistry.
**Figure S1.** Measurement of pH levels following decellularization.
**Figure S2.** Measurement of motor functional recovery rate.
**Figure S3.** Surgical procedure of animal experiment in the early stage.
**Figure S4.** Assessment of inflammatory response in the early stage.
**Figure S5.** Assessment of neovascularization and collagen deposition in the early stage.

**Movie S1.** Gait of the Native group at 24 weeks post‐implantation.

**Movie S2.** Gait of the Autograft group at 24 weeks post‐implantation.

**Movie S3.** Gait of the N‐DCN group at 24 weeks post‐implantation.

**Movie S4.** Gait of the Defect group at 24 weeks post‐implantation.

## Data Availability

The data that support the findings of this study are available from the corresponding author upon reasonable request.
