# Supplementary material for: Enhancing peripheral nerve regeneration through NaOH‐based decellularization of human nerve tissue
Source: Bioeng Transl Med. 2025 Sep 12;10(6):e70072. doi: 10.1002/btm2.70072 (PMC12617551; doi:10.1002/btm2.70072)
Supplement: Supplementary file 1 — Table S1. Primary antibodies and detection kits used in immunohistochemistry. Figure S1. Measurement of pH levels following decellularization. Figure S2. Measurement of motor functional recovery rate. Figure S3. Surgical procedure of animal experiment in the early stage. Figure S4. Assessment of inflammatory response in the early stage. Figure S5. Assessment of neovascularization and collagen deposition in the early stage. [file BTM2-10-e70072-s001.docx]

Supplementary Materials for:

**Enhancing Peripheral Nerve Regeneration Through NaOH-Based Decellularization of Human Nerve Tissue**

Subin Kim^1†^, Seong Hyuk Park^2†^, Ji Yeon Mun^1^, Soon Won Jung^1^, Won Jai Lee^2^, Dong Won Lee^2^*, Kee-Won Lee^1^*

*Corresponding author: E-mail: [xyphoss@yuhs.ac](mailto:xyphoss@yuhs.ac), [klee92@lncbio.co.kr](mailto:klee92@lncbio.co.kr)

**Contents**

**Supplementary Materials and Methods.**

**Supplementary Table S1**. **Primary Antibodies and Detection Kits Used in Immunohistochemistry**

**Supplementary Figure S1. Measurement of pH levels following decellularization.**

**Supplementary Figure S2. Measurement of motor functional recovery rate.**

**Supplementary Figure S3. Surgical procedure of animal experiment in the early stage.**

**Supplementary Figure S4. Assessment of inflammatory response in the early stage.**

**Supplementary Figure S5. Assessment of neovascularization and collagen deposition in the early stage.**

**Supplementary Movie S1. Gait of the Native group at 24 weeks post-implantation**

**Supplementary Movie S2. Gait of the Autograft group at 24 weeks post-implantation**

**Supplementary Movie S3. Gait of the N-DCN group at 24 weeks post-implantation**

**Supplementary Movie S4. Gait of the Defect group at 24 weeks post-implantation**

**Supplementary Materials and Methods.**

*Measurement of pH levels following decellularization.*

To confirm the effects of NaOH treatment and the complete removal of anions, we checked the pH levels at critical steps of decellularization process. Wash buffer samples were collected before and after NaOH treatment, as well as the final wash step. The pH levels of each sample were measured using a pH meter (SevenExcellence, Mettler-Toledo, Columbus, OH, USA).

***Animals and surgical procedures for the additional early stage in vivo experiments.***

All experimental protocols were approved by the Institutional Animal Care and Use Committee of Dt&CRO, a preclinical testing facility (Gyeonggi-do, Korea) (Approval number: DTE25-0037). All procedures adhered to the institutional ethical standards for animal research and followed the Animal Research: Reporting of In Vivo Experiments (ARRIVE) guidelines. Sixty male New Zealand White rabbits (15 weeks old, 2.5 kg; KOATECH, Korea) were randomly assigned to four experimental groups (n = 15 per group): i) excised rabbit nerve (Autograft), ii) NaOH-based decellularized human nerve (N-DCN), iii) decellularized human nerve using the Hudson method (Hudson), and ⅳ) nerve defect without treatment (Defect). An additional group of nine untreated rabbits served as the normal control (Native). The subsequent procedures were performed as described in the manuscript.

***Immunohistochemistry***

Sciatic nerve samples were longitudinally trimmed along the graft line, with one segment collected from each sample, and re-fixed in 10% neutral buffered formalin for 24 h. Paraffin blocks were then prepared using an automated tissue processor (Shandon Citadel 2000, Thermo Scientific, USA) and embedding center (Shandon Histostar, Thermo Scientific, USA). Five serial sections (4 μm thick) were obtained from each paraffin block using an automated microtome (RM2255, Leica Biosystems, Germany). Immunohistochemical staining was performed to assess the expression of CD68 (NBP2-32831, Novus Biologicals, USA), a macrophage marker; CD31 (NB600-562, Novus Biologicals), an angiogenesis marker; and collagen type I(NB600-40, Novus Biologicals). Tissue sections were incubated with purified primary antibodies (**Table S1**) and visualized using the avidin-biotin complex (ABC) method and a peroxidase substrate kit (Vector Labs, Burlingame, USA), following previously described protocols with minor modifications.^1-3^ Samples with positive staining in more than 30–40% of the epithelial or dermal regions were considered positive. longitudinally trimmed sciatic nerve tissues were examined using a light microscope (Model Eclipse 80i, Nikon, Japan), equipped with a digital camera system (ProgResTM C5, Jenoptik Optical Systems GmbH, Germany) and a computer-assisted automated image analyzer (iSolution FL ver 9.1, IMT i-solution Inc., Canada). All analyses were performed in a blinded manner with respect to group. CD68- and CD31-positive cells were quantified as the number of cells per square millimeter (cells/mm²) using automated image analysis software (*i*Solution FL ver 9.1, IMT *i*-solution Inc.,) The area of collagen type I-positive fibers was quantified as the percentage of stained area per square millimeter (%/mm²). All analyses were performed in a blinded manner with respect to group allocation.

**References**

1. Kim, D. H.; Shin, S.-H.; Lee, M.-K.; Lee, J.-J.; Kim, J. K.; Chung, Y.-G., Effectiveness and biocompatibility of decellularized nerve graft using an In vivo rat sciatic nerve model. *Tissue Engineering and Regenerative Medicine* **2021,** 18, (5), 797-805.

2. Park, S.-M.; Jung, C.-J.; Lee, D.-G.; Yu, Y.-E.; Ku, T.-H.; Hong, M.-S.; Lim, T.-K.; Paeng, K.-I.; Cho, H.-K.; Cho, I.-J., Elaeagnus umbellata fruit extract protects skin from ultraviolet-mediated photoaging in hairless mice. *Antioxidants* **2024,** 13, (2), 195.

3. Kim, E. O.; Lee, D. G.; Jung, C. J.; Yu, Y. E.; Hong, M.; Cho, I. J.; Ku, S. K., Prunus persica Leaf Extract Mitigates Ultraviolet-Induced Skin Damage Via Activation of AMP-Activated Protein Kinase. *Natural Product Communications* **2025,** 20, (4), 1934578X251330952.

**Supplementary Table**

**Table S1.** Primary Antibodies and Detection Kits Used in Immunohistochemistry

| Antibodies or detection kits | Code | Source | Dilution |
| --- | --- | --- | --- |
| CD68/SR-D1 Antibody (SPM130) | NBP2-32831 | Novus Biologicals, Centennial CO, USA | 1:100 |
| CD31/PECAM-1 Antibody (JC/70A) | NB600-562 | Novus Biologicals, Centennial CO, USA | 1:100 |
| Collagen I Antibody | NB600-408 | Novus Biologicals, Centennial CO, USA | 1:100 |
| Detection kits | | | |
| Vectastain Elite ABC kit | PK-6200 | Vector Lab., Burlingame, CA, USA | 1:50 |
| Peroxidase substrate kit | SK-4100 | Vector Lab., Burlingame, CA, USA | 1:50 |

*All antibodies were diluted using 0.01 M phosphate buffered saline (pH 7.2)

*ABC = Avidin-biotin-peroxidase complex

**Supplementary Figure**

**
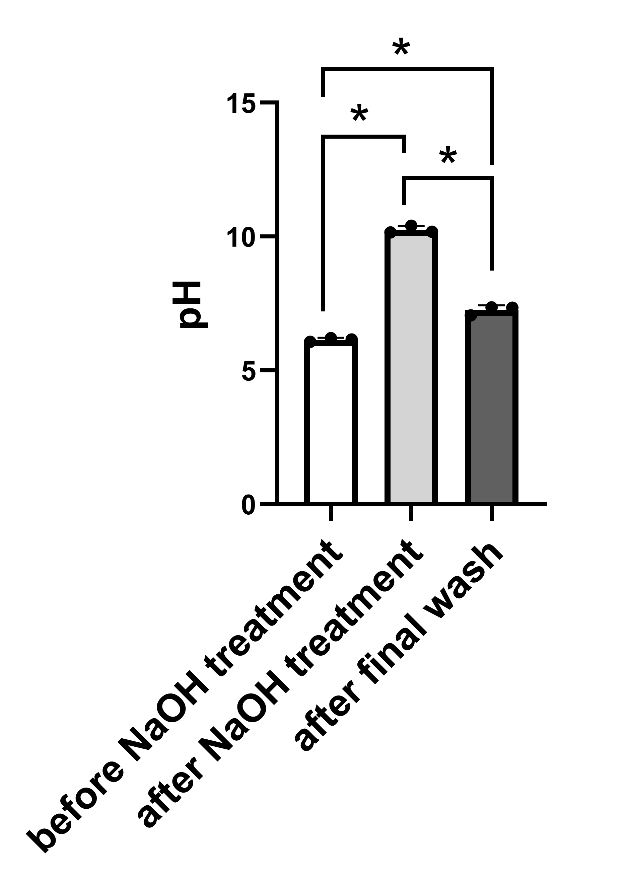
**

**Figure S1. Measurement of pH levels following decellularization.** Measurement of pH levels before, after NaOH treatment, and after wash in decellularization. Data are presented as mean ± SD (n = 3). * indicates *p* < 0.05.

**(a)**

**100% recovery**

**50% recovery**


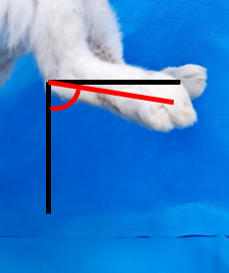

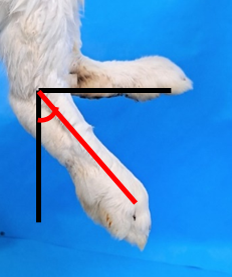

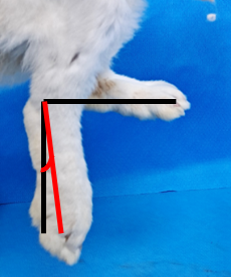


**Not recovery**


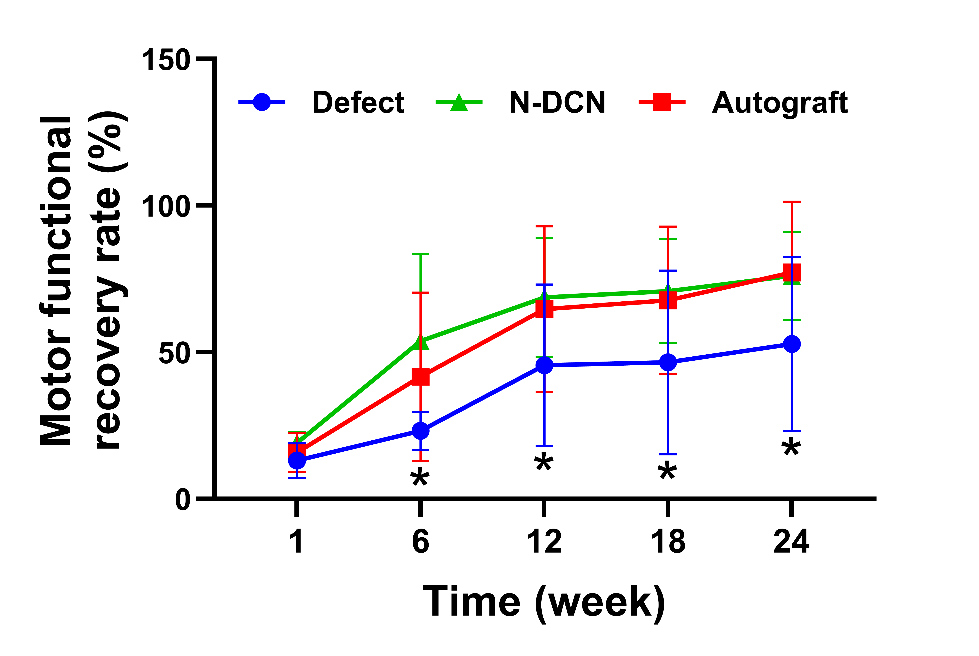


**(b)**

**Figure S2. Measurement of motor functional recovery rate.** (a) Representative images showing the motor functional recovery rate in according the angle of the rabbit’s ankle. (b) Quantification of the motor functional recovery rate of ankle in each group at 1, 6, 12, 18, and 24 weeks post-implantation. Data are presented as mean ± SD (n = 6). * indicates *p* < 0.05.

**
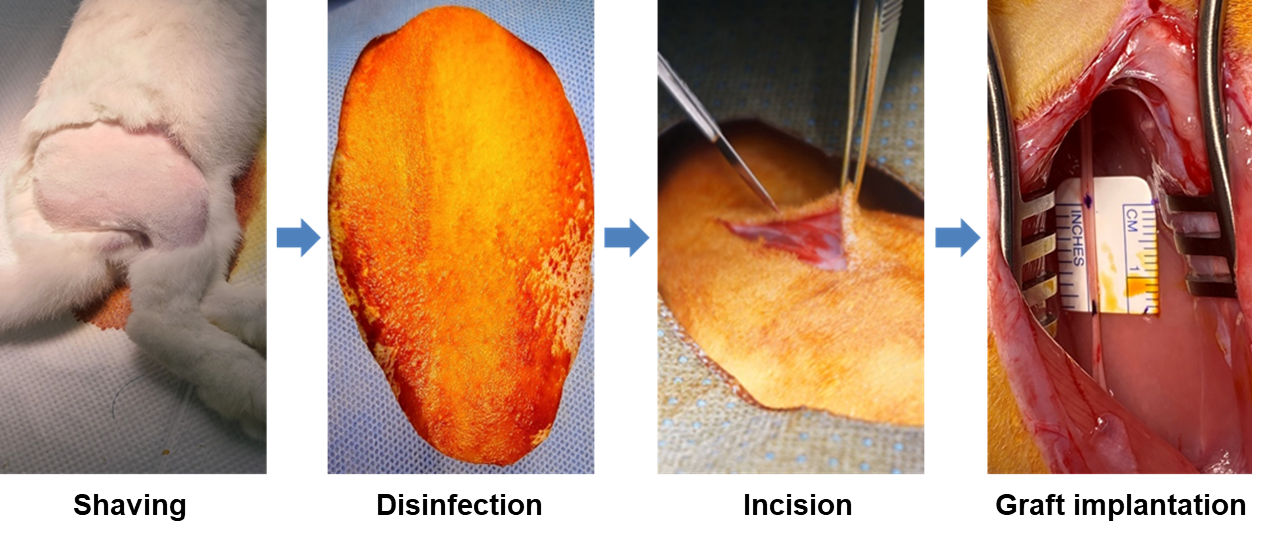
**

**Figure S3. Surgical procedure of animal experiment in the early stage of implantation.** The fur over the right hind limb was shaved and disinfected with povidone-iodine. A skin incision was then made to expose the sciatic nerve, followed by graft implantation.

**
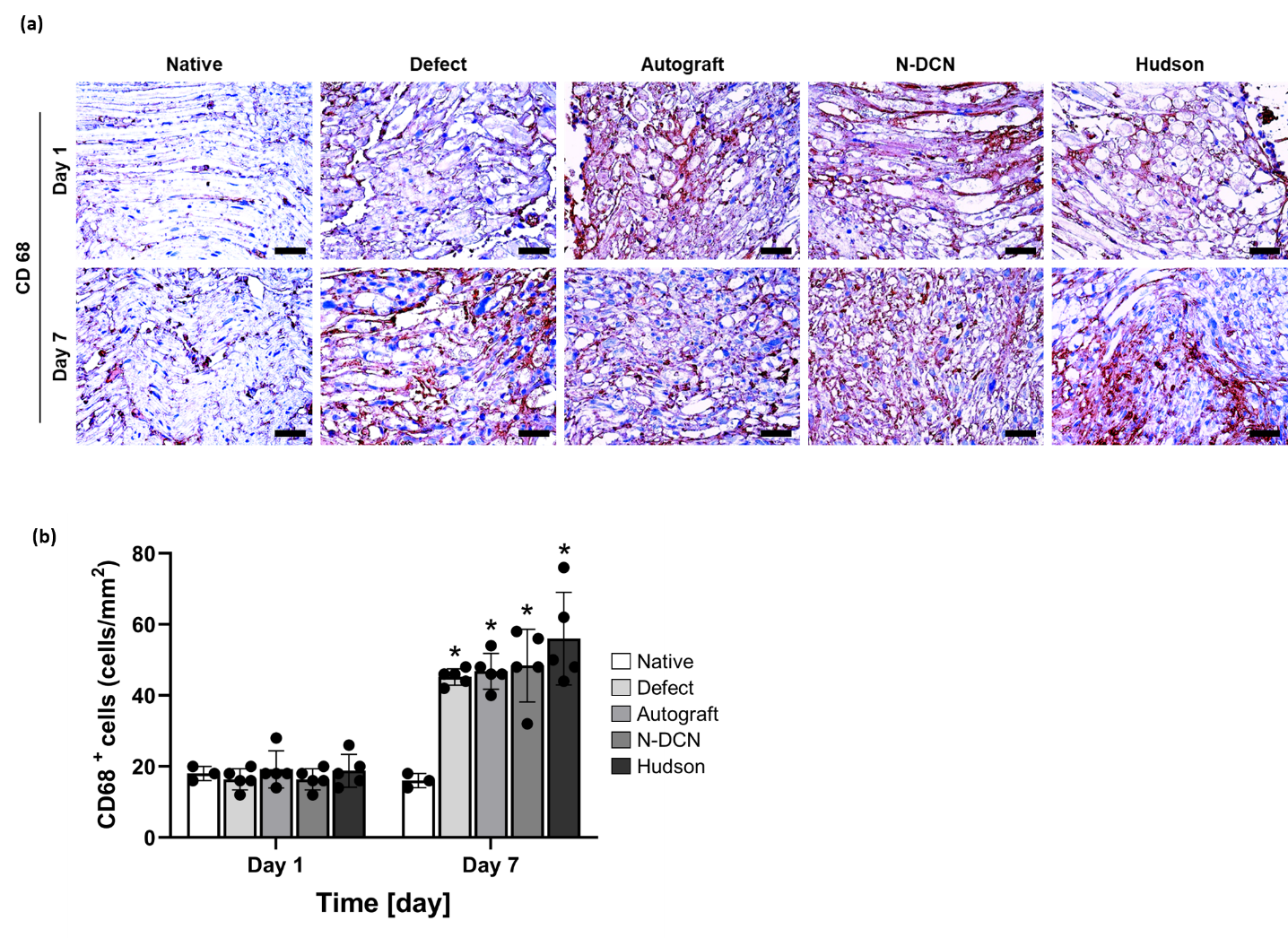
**

**Figure S4. Assessment of inflammatory response in the early stage of implantation.** (a) Representative immunohistochemical images and (b) quantification of CD68 expression in each of group at 1 and 7 days post-implantation. Magnification = 400X. Scales bars = 50 µm. Data are presented as mean ± SD (Native group : n = 3, Other groups : n = 5). * indicates *p* < 0.05 compared with the Native group.

**
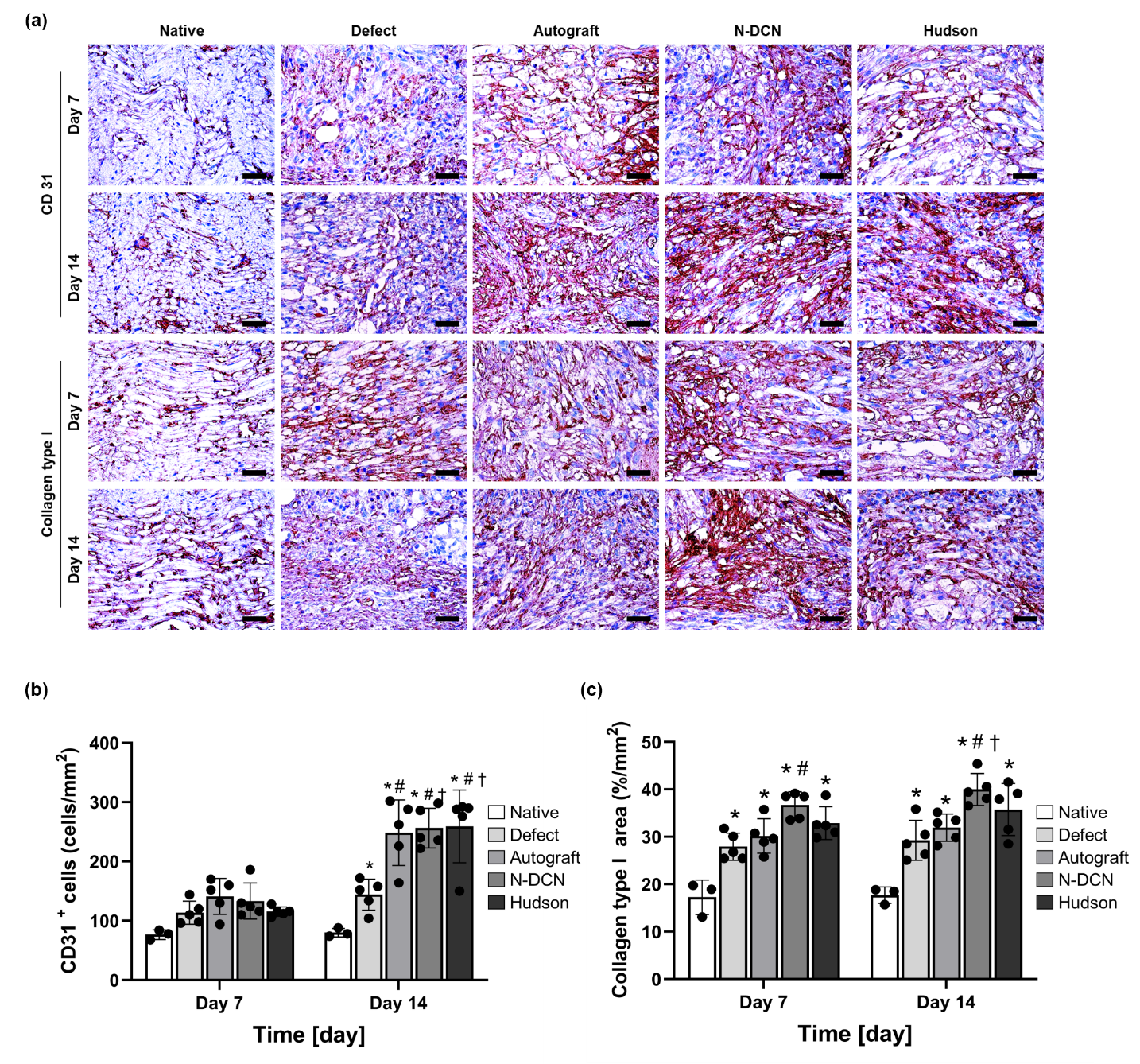
**

**Figure S5. Assessment of and neovascularization and collagen deposition in the early stage of implantation**. (a) Representative immunohistochemical images of CD31 and collagen type I, (b) quantification of CD31 expression, (c) quantification of collagen type I expression in each of group at 7 and 14 days post-implantation. Magnification = 400X. Scales bars = 50 µm. Data are presented as mean ± SD (Native group : n = 3, Other groups : n = 5). * indicates *p* < 0.05 compared with the Native group. ^#^ indicates *p* < 0.05 compared with the Defect group. ^†^ indicates *p* < 0.05 compared with the Autograft group.
